# Study of Viscoelastic Rubber Mounts on Vehicle Suspensions with In-Wheel Electric Motors

**DOI:** 10.3390/ma14123356

**Published:** 2021-06-17

**Authors:** Santiago D. Puma-Araujo, Renato Galluzzi, Xavier Sánchez-Sánchez, Ricardo A. Ramirez-Mendoza

**Affiliations:** 1 School of Engineering and Sciences, Tecnologico de Monterrey, Monterrey 64849, Mexico; ricardo.ramirez@tec.mx; 2 School of Engineering and Sciences, Tecnologico de Monterrey, Mexico City 14380, Mexico; 3 Mechatronics Laboratory, Politecnico di Torino, 10129 Turin, Italy; 4 Departamento de Ciencias de la Energía y Mecánica, Universidad de las Fuerzas Armadas ESPE, Ave. El Progreso S/N, Sangolquí 171103, Pichincha, Ecuador; xrsanchez@espe.edu.ec

**Keywords:** rubber, mount, bushing, Maxwell model, electric vehicle, in-wheel motor, vehicle suspension, mechanical vibrations, automotive engineering, vehicle dynamics

## Abstract

Rubber bushings and mounts are vastly used in automotive applications as support and interface elements. In suspension systems, they are commonly employed to interconnect the damping structure to the chassis. Therein, the viscoelastic nature of the material introduces a desirable filtering effect to reduce mechanical vibrations. When designing a suspension system, available literature often deals with viscoelastic mounts by introducing a linear or nonlinear stiffness behavior. In this context, the present paper aims at representing the rubber material using a proper viscoelastic model with the selection of different in-wheels motors. Thus, the mount dynamic behavior’s influence in a suspension is studied and discussed thoroughly through numerical simulations and sensitivity analyses. Furthermore, guidelines are proposed to orient the designer when selecting these elements.

## 1. Introduction

Bushings and mounts are compliant elements that find their application in engineering solutions as noise and vibration absorbers. Usually, they are made of flexible materials, such as rubber. They serve as an interface between rigid elements. Although bushings may be seen as support or secondary components in machinery, they are critical in guaranteeing a favorable dynamic response in these systems.

Accordingly, bushings are found in a vast range of applications. Manufacturers have paid particular attention to the dynamic properties of bushings and their influence on the resulting system dynamics. There is a wide variety of materials that can be used to manufacture bushings. The mechanical properties of these materials depend on the operating temperature ranges and the loads to which the bushings components are subject. The desired mechanical properties, namely compression strength, creep resistance, thermal expansion, and thermal conductivity, are relevant when selecting materials for a specific task. Once a material has been chosen to manufacture a rubber mount, the mechanical properties of the specimen depend not only on the material but also on the components’ geometric features.

In rotating machines, rubber mounts allow for a safe transition through critical speeds of the rotor. In the automotive industry, bushings guarantee stability by supporting subsystems, such as the internal combustion engine and the vehicle suspension links. In the latter instance, rubber elements can influence the vertical dynamics of the chassis and the unsprung masses. Moreover, the introduced dynamic characteristics can be complex because the material response depends on different variables, e.g., frequency, amplitude, preload, and temperature. This situation is even more challenging when dealing with electric vehicles (EVs) equipped with the in-wheel powertrain. In-wheel motors are direct-drive solutions. As such, they require an immediate connection to the vehicle wheel, thus yielding high-torque, low-angular-speed performance. The entire transmission system is eliminated in these solutions, with relevant advantages, such as weight reduction and no transmission friction, noise, or vibrations. However, the direct coupling between motor and wheel implies that the in-wheel solution is added to the vehicle’s unsprung mass, thus having a negative impact on vertical dynamics. Hence, matching suitable in-wheel and bushing elements is mandatory for safety, passenger comfort, and vehicle road holding.

Research on the behavior of bushings for automotive suspensions presents various contributions. Kadlowec et al. [1] proposed an experimental study that relates suspension forces and moments with their corresponding nonlinear displacement and rotation due to the bushing’s intrinsic viscoelasticity. Works from Ambrósio and Pombo [2] described a formulation in which loosely coupled bushing joints share the same kinematic information, making their modeling parameters comparable. Similarly, Ambrósio [3] verified the bushing’s vertical compliance as a function of geometry changes.

Specific studies have been carried out on EV suspension systems. Recently, Kulkarni et al. [4] compared the increased vibration in the seat of an EV compared to an internal combustion engine vehicle. For EVs, this represented a significant comfort reduction affecting the driver’s seat. Furthermore, Jin et al. [5], developed an 11-degree-of-freedom model to study the comfort of in-wheel motor vehicles while running on an ISO B class road. The work used a controlled semi-active air suspension to enhance comfort. Shi et al. [6] designed a multi-stage passive vibration isolation system. Wang et al. [7] proposed a model and mathematical method to optimize multiple dynamic parameters based on the artificial fish swarm algorithm. Liu et al. [8] developed a dynamic vibration damper that can be used for both inner rotor and outer rotor motors. It was controlled by fuzzy logic, which shows better performance in the suspension system and greater comfort. Saho et al. [9] established fuzzy control in an active system for a quarter vehicle simulation to reduce the dynamic force transmitted to the suspension elements. The study on wheeled motor vehicles is still in progress.

The state of the art shows that bushings have been extensively studied in automotive systems as a structural support of the internal combustion engine. However, the role of bushings in vehicle suspensions has been seldom addressed or neglected in the case of in-wheel motor suspensions. More importantly, when included in the vehicle suspension, the rubber mount models are often simplistic, the numerical parameters are unrealistic, or their selection is unjustified. Furthermore, as stated previously, the role of bushings is critical when increasing the unsprung mass.

In other engineering areas, research has focused on developing models and optimizing the bushing’s parameters. Bonfitto et al. [10] proposed using genetic algorithms to identify the parameters of the Maxwell–Wiechert viscoelastic model for rubber bushings used in high-speed turbomachinery. Lee et al. [11] presented a new empirical modeling method based on the Berg and Dzierzek models to identify rubber parameters. Furthermore, Ueda et al. [12] developed a model that involves the frequency and amplitude to generate a harmonic input on the studied element. Cherian et al. [13] validated a nonlinear model with its counterpart in ADAMS. García et al. [14] proposed three lumped-parameter viscoelastic models: Voigt–Kelvin, Zener, and generalized Maxwell with two, three, and five parameters, respectively. Furthermore, optimization algorithms have been applied to find bushing model parameters [8,15]. Conversely, Kang et al. [16] verified the bushing performance with a finite-element approach.

In this context, the present paper deals with the analysis of bushing dynamics for in-wheel suspensions. Unlike previous efforts, this research implements a proper bushing material model identified from experimental characterizations. With this solid model at the base, we study the impact of in-wheel motor mass and the role of the bushing in the vehicle suspension. Finally, we propose a method to size custom-made suspension mounts according to vertical dynamic requirements.

To this end, this work is structured as follows. Section 1 describes the state of the art and background of to the present research. Section 2 outlines the followed methodology and describes the used dynamic models for both rubber and vertical vehicle dynamics. In Section 3, a rubber bushing is characterized and represented through the Maxwell–Wiechert representation to obtain the parameters needed for the vertical dynamic simulations. These results serve as a tool to generate a sensitivity analysis that defines the geometry of the bushing. Finally, Section 4 concludes the work.

## 2. Materials and Methods

### 2.1. Methodology Overview

This section describes the method followed in this work, which aims at defining a custom-sized mount element by optimizing vehicle performance, while also fulfilling material and geometric constraints.

As a first instance, we define the dynamic models for the mount and the vehicle. Due to its viscoelastic nature, the mount is represented through the Maxwell–Wiechert model. On the other hand, the vehicle is assumed as a single corner that reproduces the vertical dynamics. In this regard, comfort and handling metrics are of fundamental interest as benchmark metrics. In a preliminary stage, we discuss the impact of the in-wheel motor mass, the suspension damping, and the viscoelastic mount parameters on the benchmark above variables.

**Figure 1:**
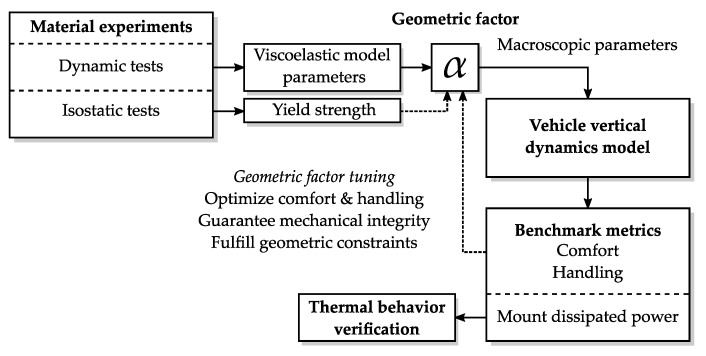
Flowchart of the proposed method.

With these tools, the methodology in Figure 1 is followed.

Firstly, the bushing material is characterized through dynamic tests and isostatic tests. Consequently, the viscoelastic parameters are obtained to fit the Maxwell–Wiechert model of the material. Thereafter, a specific bushing geometry is set through the geometric factor α, which converts the rheology parameters of the material model into macroscopic parameters of the mount element.

The selection of α aims at optimizing comfort and handling behavior of the vehicle. Nevertheless, this tuning is constrained by the mechanical integrity of the bushing, i.e., the maximum stress that it can handle. Moreover, the available space in the suspension is crucial when installing these elements, and thus, it will limit the geometric dimensions of the bushing.

After defining α, the bushing parameters can be converted from the rheologic domain (Young’s moduli and dissipation rates) to the macroscopic domain (stiffness and viscous damping coefficients). Successively, simulations of the vehicle model are necessary to evaluate the comfort and handling performance. A proper bushing sizing requires a sensitivity analysis of these benchmark variables while varying the geometric factors of the suspension bushings. Thus, multiple simulations are executed and analyzed to optimize the bushing geometry selection.

Finally, the power dissipation on the bushing is also analyzed to understand possible influences on the thermal behavior of the elements.

### 2.2. Viscoelastic Material Model

Rubber mounts and bushings are extensively used as vibration isolation due to their viscoelastic nature. Viscoelasticity yields an intrinsic hysteretic behavior, which has been addressed by numerous research works [17,18].

**Figure 2:**
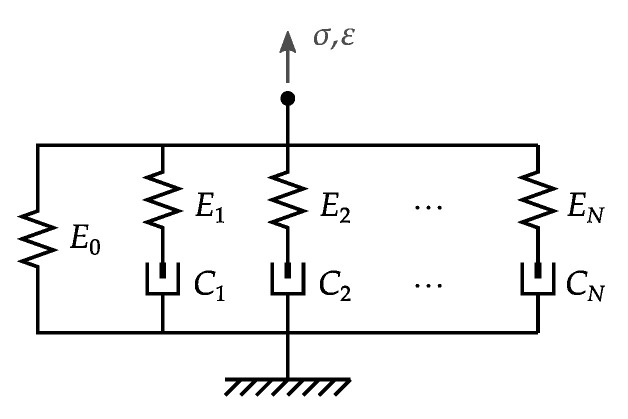
Maxwell–Wiechert viscoelastic model with elastic branch Young’s modulus $E_{0}$ and *N* viscoelastic branches characterized by $E_{i},C_{i}$.

Viscoelastic materials exhibit two main contributions: restoring stress proportional to the strain and damping stress proportional to the strain rate [19]. The former term is perfectly elastic, whereas the latter is dissipative. Both can be combined into different series/parallel arrangements, thus establishing well-known models in the literature. Among these, we can highlight the Maxwell–Wiechert model in Figure 2.

The Maxwell–Wiechert model presents a purely elastic branch denoted by Young’s modulus $E_{0}$ in parallel to *N* viscoelastic branches characterized by Young’s modulus $E_{i}$ and viscous dissipation rate $C_{i}$ in series. The presence of multiple branches is advantageous in reproducing a viscoelastic material’s dynamic behavior within a broad frequency range. In some cases, the Maxwell–Wiechert representation can be modified by including nonlinear elements that replace or enhance the purely viscous dissipation rate terms $C_{i}$.

In the particular case of vehicle applications, the frequency range of interest for vertical dynamics is usually below 100Hz. This feature allows simplifying the viscoelastic material as a Maxwell–Wiechert representation with N=1. This particular variant is known in the literature as the Kelvin model of viscoelasticity. Assuming a linear representation in the Laplace domain, the stress–strain relationship can be written as

(1) $$\begin{array}{c} \frac{\sigma (s)}{\varepsilon (s)}=E_{0}+\frac{E_{1}s}{s+E_{1}/C_{1}} \end{array}$$

where σ is the output stress, ε is the input strain, and *s* is the Laplace variable. Note that in quasi-static conditions (s→0), the material is ruled by its elastic branch $E_{0}$. By converse, as s→∞, the material tends to a stiffer behavior $E_{0}+E_{1}$. The transition dynamics among these two limit conditions are dictated by the dissipation rate coefficient $C_{1}$.

The outlined model can be used for an accurate description of the viscoelastic behavior of rubber mounts. Subsequently, these mount models can be integrated into the vertical vehicle dynamics model.

### 2.3. Vehicle Model

**Figure 3:**
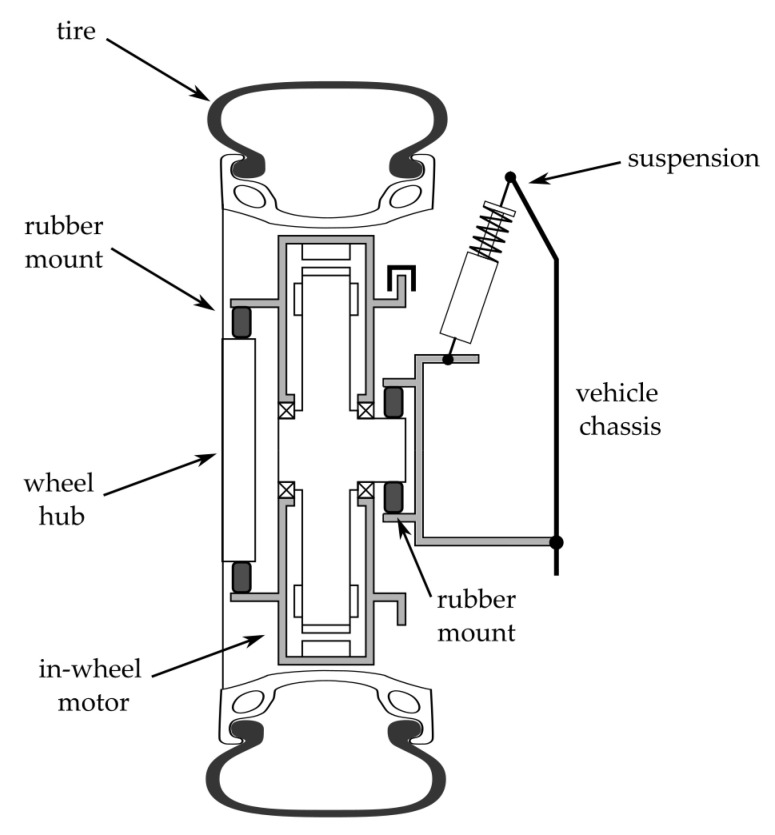
Suspension system for a vehicle equipped with in-wheel electric motor.

This research deals with the vertical dynamics of a vehicle equipped with in-wheel electric machines. In EVs, in-wheel motors are a promising alternative that requires fewer mechanical interface and transmission elements because they directly drive the wheels, thus yielding a more efficient powertrain from a mechanical perspective. Electric machines for in-wheel applications are designed to meet high-torque low-angular speed working points. They are typically brushless permanent magnet configurations with outer rotors. The wheel of the vehicle is usually coupled to the machine rotor. In contrast, the motor’s stator is linked to the vehicle suspension, which supports the vehicle chassis. The added mass associated with the electric motor demands the insertion of two rubber elements: one between the wheel hub and the rotor and another between the stator and the suspension arm. The studied configuration is schematized in Figure 3.

**Figure 4:**
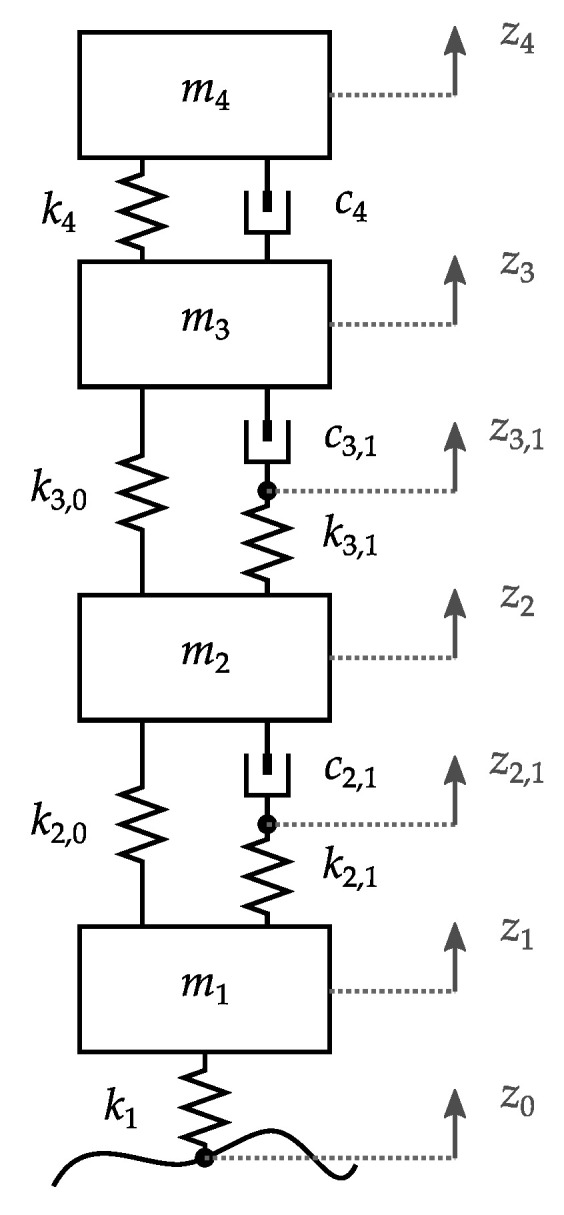
Dynamic model for vertical dynamics of a vehicle equipped with an in-wheel motor.

The dynamics of this configuration are depicted by the lumped-parameter model of Figure 4, which presents the motion over the vertical axis of one-quarter of a vehicle. The vehicle tire is represented as a stiffness $k_{1}$. The complete wheel assembly is denoted by a mass $m_{2}$. The mass $m_{2}$ represents the inertial contribution of the whole electric motor assembly. The suspension is characterized by an arm mass $m_{3}$, a suspension stiffness $k_{4}$ and a shock absorber viscous damping $c_{4}$. The chassis contribution is lumped in mass $m_{4}$.

The installation of the in-wheel motor requires rubber mount elements denoted by $k_{2,0},k_{2,1},c_{2,1}$ between the wheel and the rotor, and $k_{3,0},k_{3,1},c_{3,1}$ between the stator and the suspension arm. These stiffness and damping terms are related to the Kelvin model parameters introduced in Section 2.2. The geometric factor α is introduced to transform Young’s moduli into stiffness values and dissipation rates into viscous damping coefficients. For the generic stiffness *k* and damping *c*, we have

(2) $$\begin{array}{cc} k & =\alpha E \end{array}$$

(3) $$\begin{array}{cc} c & =\alpha C \end{array}$$

The geometric factor α is derived as the ratio of an equivalent cross-section of the bushing *A* and an equivalent axial thickness *h*:

(4) $$\begin{array}{c} \alpha =\frac{A}{h} \end{array}$$

As previously stated, the electric machine is fitted into the suspension between two rubber mounts. Since both elements can assume different geometries, dedicated factors $\alpha_{2}$ and $\alpha_{3}$ are needed.

According to the described model, equations can be established through Newton’s laws. Equations (5)–(10) denote the dynamics governing the described system.

(5) $$\begin{array}{cc} m_{1}{\ddot{z}}_{1}+k_{1}\left(z_{1}-z_{0}\right)+k_{2,0}\left(z_{1}-z_{2}\right)+k_{2,1}\left(z_{1}-z_{2,1}\right) & =0 \end{array}$$

(6) $$\begin{array}{cc} c_{2,1}\left({\dot{z}}_{2,1}-{\dot{z}}_{2}\right)+k_{2,1}\left(z_{2,1}-z_{1}\right) & =0 \end{array}$$

(7) $$\begin{array}{cc} m_{2}{\ddot{z}}_{2}+c_{2,1}\left({\dot{z}}_{2}-{\dot{z}}_{2,1}\right)+k_{2,0}\left(z_{2}-z_{1}\right)+k_{3,0}\left(z_{2}-z_{3}\right)+k_{3,1}\left(z_{2}-z_{3,1}\right) & =0 \end{array}$$

(8) $$\begin{array}{cc} c_{3,1}\left({\dot{z}}_{3,1}-{\dot{z}}_{3}\right)+k_{3,1}\left(z_{3,1}-z_{2}\right) & =0 \end{array}$$

(9) $$\begin{array}{cc} m_{3}{\ddot{z}}_{3}+c_{3,1}\left({\dot{z}}_{3}-{\dot{z}}_{3,1}\right)+c_{4}\left({\dot{z}}_{3}-{\dot{z}}_{4}\right)+k_{3,0}\left(z_{3}-z_{2}\right)+k_{4}\left(z_{3}-z_{4}\right) & =0 \end{array}$$

(10) $$\begin{array}{cc} m_{4}{\ddot{z}}_{4}+c_{4}\left({\dot{z}}_{4}-{\dot{z}}_{3}\right)+k_{4}\left(z_{4}-z_{3}\right) & =0 \end{array}$$

Following the notation in Figure 4, Equations (5)–(10) are solved for the generalized coordinates that describe the vertical dynamic evolution of the four mass elements ($z_{1\ldots 4}$) and the interconnection points in the viscoelastic branches of the rubber elements ($z_{2,1},z_{3,1}$).

#### 2.3.1. Road Profile

The input of the described system is the road unevenness, i.e., the vertical displacement $z_{0}$. This input can be synthesized according to the ISO 8608 standard [20], which defines the power spectral density (PSD) of $z_{0}$ as

(11) $$\begin{array}{c} S_{0}=\frac{G_{r}V}{f^{2}} \end{array}$$

where *V* is the longitudinal speed, and $G_{r}$ denotes the road roughness index, a constant that depends on the road class. ISO defines letter-designated road qualities employing different $G_{r}$ coefficients. The road PSD also depends on the time-frequency *f*, as the behavior of $|S_{0}|$ in the frequency domain exhibits an attenuation of 40dB/dec.

The PSD in Equation (11) can be synthesized with a unit-power white noise signal filtered by a first order low-pass transfer function [21]

(12) $$\begin{array}{cc} H_{8608}\left(s\right) & =\frac{2\pi \sqrt{G_{r}V}}{s+2\pi f_{0}} \end{array}$$

with a filter cutoff frequency $f_{0}=V/\lambda_{0}$ characterized by a sufficiently large wavelength $\lambda_{0}≅100\;m$.

#### 2.3.2. Outputs of Interest

In vehicle dynamics there are two main metrics that denote the ride quality: comfort and road holding.

Comfort is associated with the sensitivity of the human body when absorbing vibrations. Although comfort is subjective, the ISO 2631 standard [22] specifies different ranges of root-mean-square vertical acceleration that the passenger may tolerate. Accordingly, the evaluation of comfort requires the calculation of the chassis acceleration ${\ddot{z}}_{4}$. Besides, ISO also defines the frequency response of human sensitivity to vibrations. This definition has been converted into a band-pass filtering function in the Laplace domain [23]:

(13) $$\begin{array}{c} H_{2631}\left(s\right)=\frac{80.03s^{2}+989s+0.02108}{s^{3}+78.92s^{2}+2412s+5614} \end{array}$$

This filtering function can be applied to ${\ddot{z}}_{4}$ to obtain a weighted acceleration response $a_{w}$ useful to assess human comfort.

Road holding denotes the vehicle’s ability to follow the road profile without losing contact from the ground. The ratio between the tire force and the weight of the vehicle *W* is a meaningful way to represent this situation:

(14) $$\begin{array}{cc} \eta_{r} & =\frac{k_{1}\left(z_{1}-z_{0}\right)}{W} \end{array}$$

(15) $$\begin{array}{cc} W & =\sum_{i=1}^{4}m_{i}g \end{array}$$

Whenever the $\eta_{r}$ ratio exceeds the unit, the dynamic force of the tire exceeds the weight of the vehicle, thus leading to a loss of contact from the road.

#### 2.3.3. State-Space Representation

The simplest way to solve for the dynamics of a system is the state-variable approach. This is a matrix representation used to describe system dynamics in a compact, standard notation. The concept of state was first introduced by Kalman in 1960 [24]. A state is a variable used to describe dynamic evolution in time.

The outlined differential equation set (Equations (5)–(10)) can be arranged into the state-space form

(16) $$\begin{array}{cc} \dot{x} & =Ax+Bu \end{array}$$

(17) $$\begin{array}{cc} y & =Cx+Du \end{array}$$

where the following vectors denote the states, inputs and outputs, respectively

(18) $$\begin{array}{cc} x & ={\left\{\begin{array}{cccccccccc} z_{1} & z_{2,1} & z_{2} & z_{3,1} & z_{3} & z_{4} & {\dot{z}}_{1} & {\dot{z}}_{2} & {\dot{z}}_{3} & {\dot{z}}_{4} \end{array}\right\}}^{⊺} \end{array}$$

(19) $$\begin{array}{cc} u & =z_{0} \end{array}$$

(20) $$\begin{array}{cc} y & ={\left\{\begin{array}{cc} {\ddot{z}}_{4} & \eta_{r} \end{array}\right\}}^{⊺} \end{array}$$

and the state, input, output and input-output mapping matrices are given by

(21) $$\begin{array}{cc} A & =\left[\begin{array}{cccccccccc} 0 & 0 & 0 & 0 & 0 & 0 & 1 & 0 & 0 & 0\\ \frac{k_{2,1}}{c_{2,1}} & -\frac{k_{2,1}}{c_{2,1}} & 0 & 0 & 0 & 0 & 0 & 1 & 0 & 0\\ 0 & 0 & 0 & 0 & 0 & 0 & 0 & 1 & 0 & 0\\ 0 & 0 & \frac{k_{3,1}}{c_{3,1}} & -\frac{k_{3,1}}{c_{3,1}} & 0 & 0 & 0 & 0 & 1 & 0\\ 0 & 0 & 0 & 0 & 0 & 0 & 0 & 0 & 1 & 0\\ 0 & 0 & 0 & 0 & 0 & 0 & 0 & 0 & 0 & 1\\ -\frac{k_{1}+k_{2,0}+k_{2,1}}{m_{1}} & \frac{k_{2,1}}{m_{1}} & \frac{k_{2,0}}{m_{1}} & 0 & 0 & 0 & 0 & 0 & 0 & 0\\ \frac{k_{2,0}+k_{2,1}}{m_{2}} & -\frac{k_{2,1}}{m_{2}} & -\frac{k_{2,0}+k_{3,0}+k_{3,1}}{m_{2}} & \frac{k_{3,1}}{m_{2}} & \frac{k_{3,0}}{m_{2}} & 0 & 0 & 0 & 0 & 0\\ 0 & 0 & \frac{k_{3,0}+k_{3,1}}{m_{3}} & -\frac{k_{3,1}}{m_{3}} & -\frac{k_{3,0}+k_{4}}{m_{3}} & \frac{k_{4}}{m_{3}} & 0 & 0 & -\frac{c_{4}}{m_{3}} & \frac{c_{4}}{m_{3}}\\ 0 & 0 & 0 & 0 & \frac{k_{4}}{m_{4}} & -\frac{k_{4}}{m_{4}} & 0 & 0 & \frac{c_{4}}{m_{4}} & -\frac{c_{4}}{m_{4}} \end{array}\right] \end{array}$$

(22) $$\begin{array}{cc} B & ={\left[\begin{array}{cccccccccc} 0 & 0 & 0 & 0 & 0 & 0 & \frac{k_{1}}{m_{1}} & 0 & 0 & 0 \end{array}\right]}^{⊺} \end{array}$$

(23) $$\begin{array}{cc} C & =\left[\begin{array}{cccccccccc} 0 & 0 & 0 & 0 & \frac{k_{4}}{m_{4}} & -\frac{k_{4}}{m_{4}} & 0 & 0 & \frac{c_{4}}{m_{4}} & -\frac{c_{4}}{m_{4}}\\ \frac{k_{1}}{W} & 0 & 0 & 0 & 0 & 0 & 0 & 0 & 0 & 0 \end{array}\right] \end{array}$$

(24) $$\begin{array}{cc} D & ={\left[\begin{array}{cc} 0 & -\frac{k_{1}}{W} \end{array}\right]}^{⊺} \end{array}$$

Studies in the frequency domain require the computation of the transfer function among the defined input and the outputs of interest. The transfer function matrix can be computed as

(25) $$\begin{array}{cc} G & =\left[\begin{array}{c} G_{1}\left(s\right)\\ G_{2}\left(s\right) \end{array}\right]=\left[\begin{array}{c} \frac{s^{2}z_{4}\left(s\right)}{z_{0}\left(s\right)}\\ \frac{\eta_{r}\left(s\right)}{z_{0}\left(s\right)} \end{array}\right]=C{\left(sI-A\right)}^{-1}B+D \end{array}$$

where I is a 4×4 identity matrix.

## 3. Results

### 3.1. Model Parameters

To illustrate the methodology described in Section 2, the vehicle model is based on the parameters listed in Table 1. These parameters belong to the reference class-A vehicle from the simulation software CarSim^TM^ 2021.0 [25], which represent a standard for the automotive industry.

At this point, the model still requires the definition of the following parameters:

- Electric motor mass $m_{2}$, which can be identified from available in-wheel powertrain solutions, but can have a negative impact on vertical dynamics.
- Suspension damping $c_{4}$, that can be tuned to guarantee suitable comfort and handling performances.
- Rubber mount parameters $k_{2,0},k_{2,1},c_{2,1}$ and $k_{3,0},k_{3,1},c_{3,1}$ which are set by following the identification of the Kelvin model from experimental data and the tuning of geometric factors $\alpha_{2}$ and $\alpha_{3}$, respectively.

These points will be analyzed in detail in the following subsections.

### 3.2. Sensitivity to In-Wheel Motor Mass and Suspension Damping

Since the reference example does not contain an in-wheel powertrain, state-of-the-art in-wheel solutions from Elaphe^TM^ [26] were analyzed (see Table 2). Among them, the S400 model is particularly lightweight and suitable for compact vehicle applications, as reported by the manufacturer.

**Figure 5:**
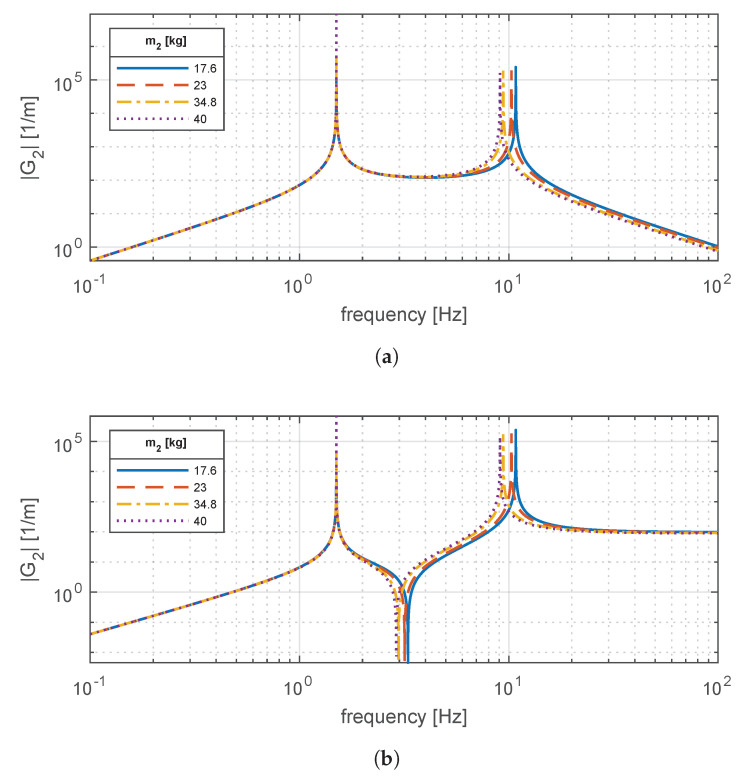
Magnitude frequency response of (**a**) *G*_1_(*s*) for comfort and (**b**) *G*_2_(*s*) for road holding for different in-wheel motor masses *m*_2_.

Figure 5 illustrates the frequency response of transfer functions $G_{1}\left(s\right)$ and $G_{2}\left(s\right)$, which describe the comfort and handling performances of the vehicle. These responses are calculated for the four in-wheel motor masses present in Table 2. In addition, the suspension damping $c_{4}$ is set to zero to analyze the undamped response, and the rubber mounts that constrain the electric machine are set as infinitely rigid ($k_{2,0}\to \infty ,k_{3,0}\to \infty$). From both transfer functions, two resonant peaks are advisable for the so-called sprung ($m_{4}$) and unsprung ($m_{1}+m_{2}+m_{3}$) masses. Due to their separation in the spectrum, it is seen that the variation of $m_{2}$ only affects the second peak.

Vibration mode frequencies can be computed by assuming the natural response of the system. Thus, forcing input signals are neglected:

(26) $$\begin{array}{c} \dot{x}=\mathrm{Ax} \end{array}$$

and frequencies are computed by solving

(27) $$\begin{array}{c} \det\left(sI-A\right)=0 \end{array}$$

However, due to the observed uncoupling, frequencies can be approximated as

(28) $$\begin{array}{cc} f_{s} & ≅\frac{1}{2\pi }\sqrt{\frac{k_{4}}{m_{4}}} \end{array}$$

(29) $$\begin{array}{cc} f_{u} & ≅\frac{1}{2\pi }\sqrt{\frac{k_{1}+k_{4}}{m_{1}+m_{2}+m_{3}}} \end{array}$$

For the sprung and unsprung frequencies, respectively. Numerically, $f_{s}≅1.56\;\mathrm{Hz}$, whereas $f_{u}$ ranges from 9.07Hz for the heaviest motor to 10.8Hz for the lightest one. Interestingly, a suspension without an in-wheel motor behaves at a higher frequency $f_{u}≅13.15\;\mathrm{Hz}$, thus highlighting the negative impact of introducing an in-wheel electric powertrain. For the proposed vehicle powertrain, the lightest machine—the S400 model—is sufficient for the task.

**Figure 6:**
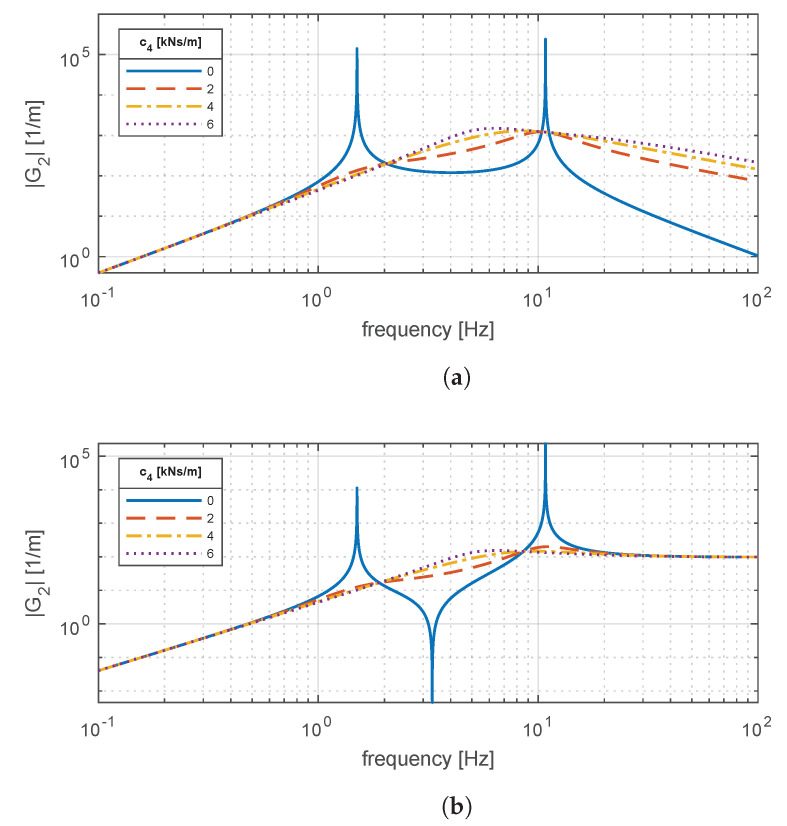
Magnitude frequency response of (**a**) *G*_1_(*s*) for comfort and (**b**) *G*_2_(*s*) for road holding for different suspension damping coefficients *c*_4_.

Figure 6 depicts the effects of varying the suspension damping on the comfort and handling responses. For comfort, it is seen that the shock absorber tends to damp the resonant peaks, thus reducing the vertical acceleration experienced by the passenger. However, the introduction of damping also tends to amplify $|G_{1}|$ within the frequency range $f_{s}\leq f\leq f_{u}$. A similar situation is observed for handling. However, in this latter case, the shape of $|G_{2}|$ is different from $|G_{1}|$. This implies that a trade-off among these two metrics needs to be solved when selecting an appropriate suspension damping. The intrinsic contradiction between comfort and handling is well-known in the literature [27].

To further explore the influence of the motor mass and the suspension damping, vertical vehicle dynamics are also assessed in the time domain for different values of $m_{2}$ and $c_{4}$. With the method presented in Section 2.3.1, the vehicle is subject to ISO C-class road irregularities while navigating at 46.5km/h, i.e., the average of the Worldwide harmonized Light vehicles Test Cycle. Root-mean-square (RMS) values of the ISO 2631-filtered chassis acceleration $a_{w}$ and road holding index $\eta_{r}$ were extracted for ten-second runs.

**Figure 7:**
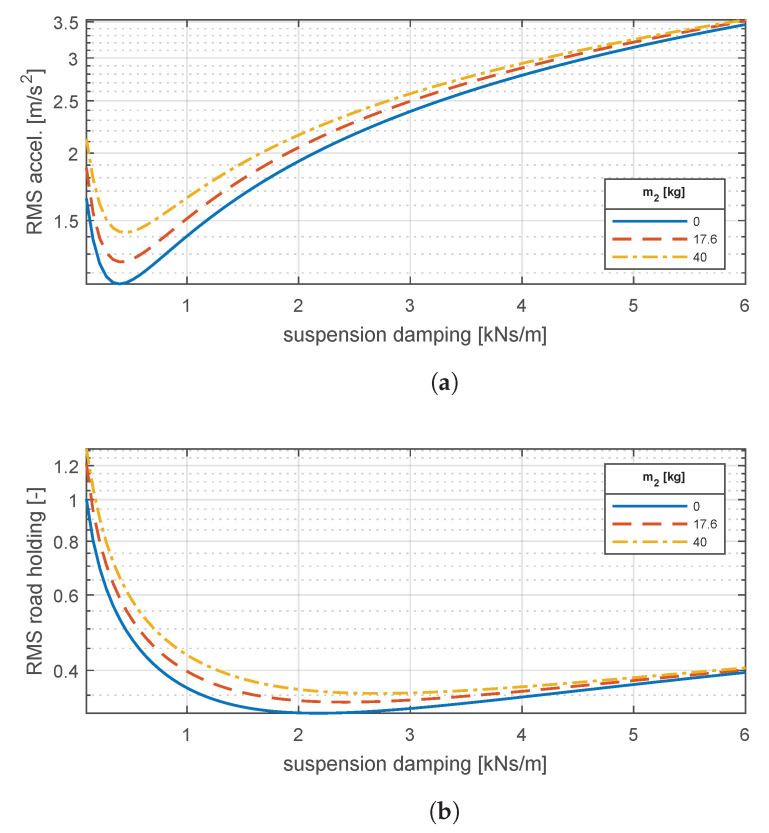
Time-domain response of vehicle vertical dynamics: (**a**) comfort response and (**b**) road holding response. Sensitivity to suspension damping (*c*_4_) and in-wheel motor mass (*m*_2_) variations.

The plots in Figure 7 illustrate the well-known tradeoff between comfort and handling tuning the suspension damping. Optimal comfort—minimum RMS chassis acceleration—is attained at relatively low values of damping. In contrast, optimal handling—minimum RMS road holding index—is reached with larger suspension damping values. The added in-wheel motor mass introduces a relevant contribution to the system dynamics. At optimal comfort, RMS chassis acceleration increases from $1.15\;m/s^{2}$ with no in-wheel motor mass up to $1.43\;m/s^{2}$ (+24.3%) with a 40kg motor mass. Likewise, RMS road holding is also negatively affected by the mass addition: from 0.354 to 0.38 (+7.3%) with the same unsprung mass increase.

For our baseline configuration with $m_{2}=17.6\;\mathrm{kg}$, optimal comfort is guaranteed with a suspension damping $c_{4}=461\;\mathrm{Ns}/m$, whereas $c_{4}=2388\;\mathrm{Ns}/m$ yields optimal road holding. For subsequent calculations, we will use this latter value to favor ride safety.

### 3.3. Material Experiments

This section details the experiments carried out to characterize statically and dynamically a bushing that could be used for suspension mounts. The material of this element is vulcanized carbon black-filled rubber, which is well-known due to its viscoelastic behavior. More specific details about the used mixture are not available, as the material is part of a commercial spare bushing. However, Hu et al. use a similar material for dynamic characterization purposes [28].

#### 3.3.1. Isostatic Tests

Standard ASTM D412 characterized the bushing material in a tensile test to determine its ultimate strength. Five samples of $28\times 5\times 4\;{\mathrm{mm}}^{3}$ are tested until failure on a Shimadzu AGS-X^TM^ (Shimadzu Corporation, Kyoto, Japan) test machine. A minimum tensile strength of 5.34MPa is identified. Among tests, a standard deviation of 0.31MPa is found.

#### 3.3.2. Dynamic Tests

Dedicated tests are carried out to identify the bushing parameters for later use in dynamic simulations. A material sample was extracted from a standard silentblock rubber bushing for automotive applications. The final sample shape is a cylinder with 6mm in diameter by 3mm of thickness. For the experiments, Discovery HR 2^TM^ hybrid rheometer (Waters Corporation, New Castle, DE, USA) is set to a minimum oscillation force of 0.03N and a maximum axial force of 50N. The device is able to record with a displacement resolution of 20nm in a range from 0.1 up to 16Hz. A parallel plate compression accessory was used to apply cyclic loading to the sample with a preload of 15N. The amplitude is established at 100μm to determine the storage ($E^{\prime }$) and loss ($E^{\prime \prime }$) moduli at different frequencies (*f*):

(30) $$\begin{array}{c} \frac{\sigma_{exp}\left(f\right)}{\varepsilon_{exp}\left(f\right)}=E^{\prime }\left(f\right)+jE^{\prime \prime }\left(f\right) \end{array}$$

**Figure 8:**
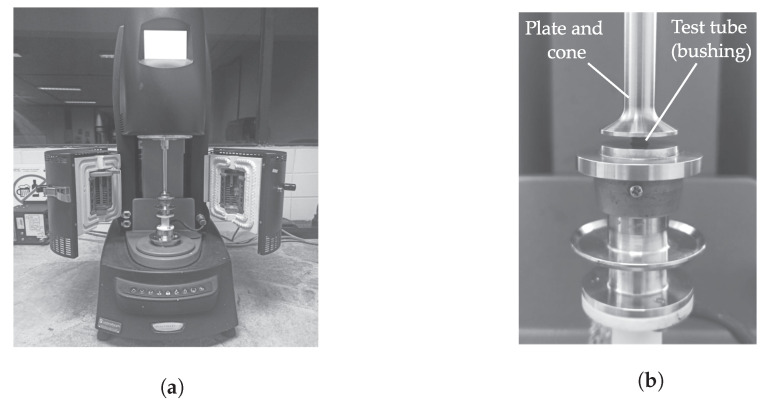
Pictures of the experimental setup (**a**) Discovery HR 2^TM^ hybrid rheometer; (**b**) Sample installed in the testing machine.

Figure 8 presents pictures of the described experimental setup. The test was carried out under the ASTM D-5024 standard at ambient temperatures ranging from 20, to $100{\;}^{{}^{\circ}}C$ and relative humidity of 30%. The thickness reduction due to the bushing measurements is accounted for. For the test, it has been considered that at frequencies lower than 0.1Hz, the excitation is quasi-static, i.e., the increase of effort is insufficient to study the dependence of the moduli as a function of frequency. By converse, the maximum frequency attained is limited by the machine capability.

Experimental results are described as magnitude and phase from the complex representation in Equation (30):

(31) $$\begin{array}{cc} \left|E(f)\right| & =\sqrt{E^{\prime }{\left(f\right)}^{2}+E^{\prime \prime }{\left(f\right)}^{2}} \end{array}$$

(32) $$\begin{array}{cc} \phi (f) & =\arctan\left(\frac{E^{\prime \prime }\left(f\right)}{E^{\prime }\left(f\right)}\right) \end{array}$$

**Figure 9:**
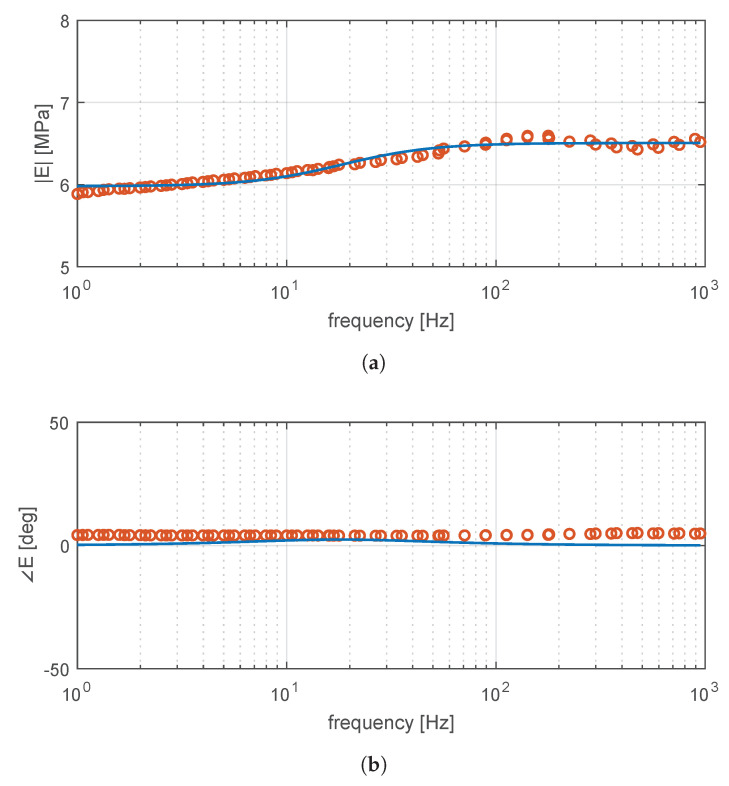
Experimental and fitted rheologic behavior of the tested rubber specimen: (**a**) magnitude and (**b**) phase. Markers indicate experiment extrapolation using the time-temperature superposition at 100 °C (circle). A fitted response using the Maxwell–Wiechert model is also shown (solid).

Following dynamic tests at different temperatures, the time-temperature superposition principle is applied to build a master curve of the material at $100{\;}^{{}^{\circ}}C$ [29,30]. Moreover, at this temperature, the frequency behavior of the elastic and loss moduli presents low variability and therefore, still far away from the glass transition temperature $T_{g}$ [28]. The obtained master curve appears in Figure 9. In dynamic conditions, high-temperature parameters are more conservative for the design, as they will allow larger displacements and greater power dissipation.

The general behavior of the magnitude and phase as a function of frequency presents a decrease in the response of the oscillation force. This can be attributed to the Payne effect [31], which appears in reinforced rubbers. In fact, the increase in the rubber moduli is due to friction effects at low frequencies produced by the vibration that, when increasing, breaks weak van der Waals bonds.

Figure 9 also shows the identification results of the Maxwell–Wiechert model parameters. The frequency response of Equation (1) is fitted with experimental amplitude and phase data.

For each measurement, an error function *e* is defined:

(33) $$\begin{array}{c} e=\left|\frac{\sigma (f)}{\varepsilon (f)}\right|-\left|\frac{\sigma_{exp}\left(f\right)}{\varepsilon_{exp}\left(f\right)}\right| \end{array}$$

Therefore, a RMS error can be computed with respect to *N* experimental samples:

(34) $$\begin{array}{c} e_{rms}=\sqrt{\frac{1}{N}\sum_{i=1}^{N}e_{i}^{2}} \end{array}$$

The RMS error can be used as a cost function for a minimization algorithm. We used a trust-region constrained minimization in MATLAB^TM^ to tune the parameters $E_{0},E_{1},C_{1}$ of the Maxwell–Wiechert model.

The obtained results are listed in Table 3. It is worth noting that the material exhibits a an elastic behavior predominantly, with viscoelastic high-pass action starting at $E_{1}/C_{1}=117.86\;\mathrm{rad}/s$, or 18.76Hz. These parameters are subsequently used for the sizing of the bushing.

### 3.4. Rubber Bushing Influence on Vertical Dynamics

**Figure 10:**
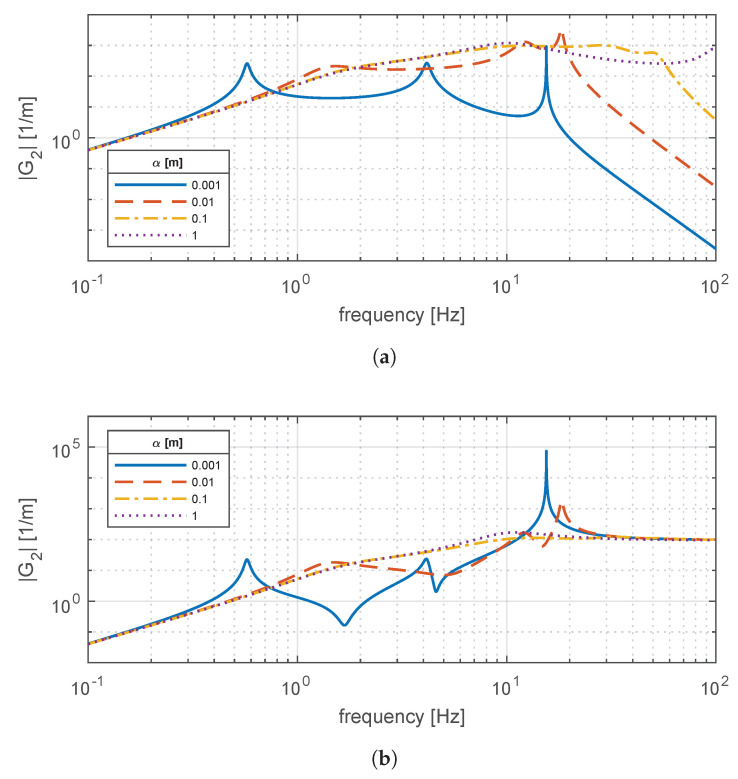
Magnitude frequency response of (**a**) *G*_1_(*s*) for comfort and (**b**) *G*_2_(*s*) for road holding for different geometry factor values *α* = *α*_2_ = *α*_3_.

Once that the rheology parameters are known ($E_{0},E_{1},C_{1}$), they can be transformed into macroscopic variables by means of the geometric factor through Equations (2) and (3). Thus, stiffness $k_{i,0},k_{i,1}$ and damping $c_{i,1}$ are obtained for i=2,3. In Figure 10, the vertical dynamics response is plotted for comfort and handling transfer functions while varying the geometric factor $\alpha =\alpha_{2}=\alpha_{3}$. In particular, the values of α range from 0.001m to 1m. Recalling Equation (4), high values of α imply large equivalent cross sections and low axial thickness, thus leading to stiff dynamic response. Conversely, low values would lead to thicker elements with reduced cross section and hence, larger mount compliance.

It is seen that the reduction of the geometric factor leads to compliant mounts that, in general, tend to favor comfort due to strong attenuation. Nevertheless, resonance regions arise due to the dynamic interaction of the mount elasticity and the supported mass (in this case, dominated by the in-wheel motor). For road holding, the attenuation benefits seen for comfort are less evident, although still present. Conversely, resonant phenomena affect more heavily the handling response.

The responses in the frequency domain confirm that the addition of mounts is necessary to improve the comfort response, which is hindered by the inclusion of the in-wheel mass. However, a more realistic simulation is needed to determine the adequate sizing of bushing elements. In addition, the frequency responses in Figure 10 assumes that both bushings supporting the mass $m_{2}$ are identical.

To address these issues, a sensitivity analysis is carried out for the suspension dynamics while varying geometric factors $\alpha_{2}$ and $\alpha_{3}$. Instead of relying in frequency response functions, the analysis is carried out in the time domain with a realistic ISO C road profile run at 46.5km/h as input. Comfort is evaluated through the ISO-filtered RMS chassis acceleration, while the RMS road holding index denotes handling.

**Figure 11:**
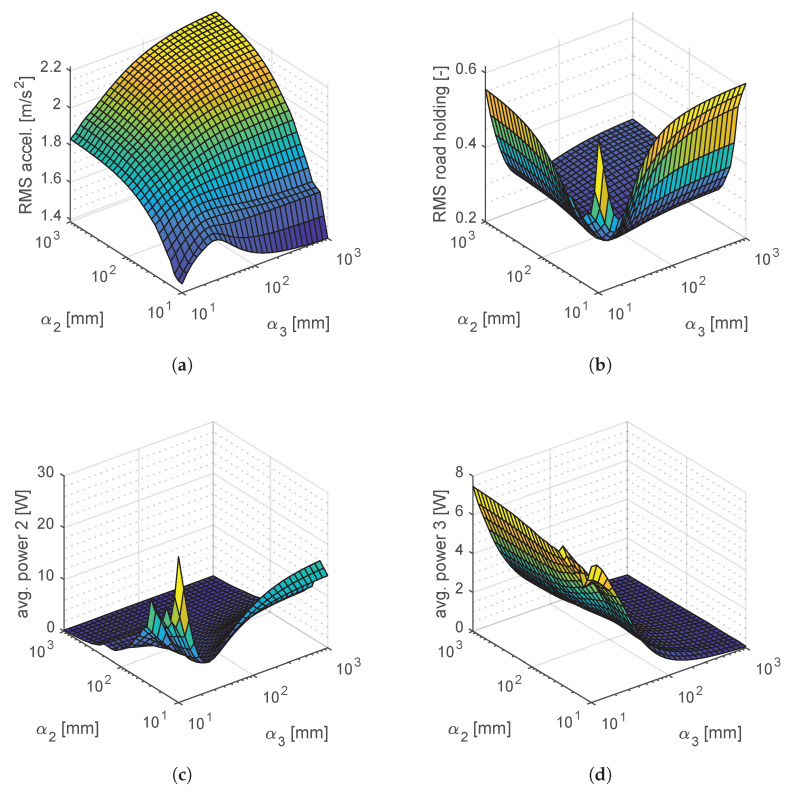
Time-domain response of (**a**) vehicle comfort, (**b**) vehicle road holding, (**c**) bottom bushing power dissipation and (**d**) top bushing power dissipation. Sensitivity to bushing geometric factors *α*_2_ and *α*_3_.

Figure 11 shows sensitivity analysis results obtained for values of $\alpha_{2}$ and $\alpha_{3}$ ranging from 10mm to 1m. In particular, comfort, handling and power dissipation on each bushing are reported while varying the geometric factor for the bottom bushing ($\alpha_{2}$) and the top bushing ($\alpha_{3}$) that support the in-wheel motor. In comfort and handling plots, it is observed that sizing both bushings similarly with $\alpha_{2}≅\alpha_{3}$ is a proper choice. For comfort, the parametric variation in the explored region shows that low geometric factors favor RMS chassis acceleration. In handling, the obtained plot is convex and presents minimum RMS road holding at $\alpha_{2}=\alpha_{3}=48.94\;\mathrm{mm}$. Recalling that α is the ratio between the bushing equivalent cross-section and its axial thickness, we establish the minimum cross-section from the ultimate tensile strength. The axial force transmitted by the bushings is calculated on every iteration as

(35) $$\begin{array}{cc} F_{2} & =\left|k_{2,0}\left(z_{1}-z_{2}\right)+k_{2,1}\left(z_{1}-z_{2,1}\right)\right| \end{array}$$

(36) $$\begin{array}{cc} F_{3} & =\left|k_{3,0}\left(z_{2}-z_{3}\right)+k_{3,1}\left(z_{2}-z_{3,1}\right)\right| \end{array}$$

and thus, the minimum cross section is defined:

(37) $$\begin{array}{c} A_{i}=\frac{F_{i}}{\sigma_{max}}\;\forall \;i=2,3 \end{array}$$

Considering a safety factor of 2, minimum bushing cross-sections $A_{2}=590.33\;{\mathrm{mm}}^{2}$ and $A_{3}=565.03\;{\mathrm{mm}}^{2}$ are identified. However, when considering limit operations, the vehicle suspension can reach forces up to 10kN in transient maneuvers. Hence, equivalent cross sections of at least $20\;{\mathrm{cm}}^{2}$ are expected. In turn, this area allows defining the mount thickness value $h_{2}=h_{3}=40.87\;\mathrm{mm}$ to match $\alpha_{2}=\alpha_{3}=48.94\;\mathrm{mm}$.

Sensitivity results in Figure 11 demonstrate that the optimal tuning of the geometric factors yield improved comfort and handling performances. This enhancement can be quantified as a reduction of 15.78% on the RMS chassis acceleration and 24.66% on the RMS road holding index when compared to the system without suspension mounts.

When determining the bushing size, one must also consider the maximum power that the bushing can dissipate as heat. For this latter aspect, Figure 11 also shows the power dissipated by bushings (labeled as i=2 and i=3) during the sensitivity analysis. The dissipated power exhibits a hyperbolic behavior that rapidly escalates as the geometric factor decreases. In the optimized condition, the average dissipated power of each bushing does not exceed 2.3W. In general, this indication would not require additional thermal analyses. However, for harsh terrain profiles, as in off-road applications, this aspect would require additional attention.

With the obtained results, it is possible to generalize the following selection guidelines for vehicle suspensions:

1. Minimize unsprung mass contributions, i.e., optimize the in-wheel motor design to yield the most lightweight solution.
2. After selecting the bushing material, provide a design with an optimal geometric factor able to minimize the RMS road holding index.
3. Determine the bushing equivalent cross section by accounting for the ultimate stress of the material.
4. Determine the bushing equivalent axial thickness needed to match the geometric factor and the equivalent cross section.
5. Verify that the design complies with the geometric constraints of the suspension. If geometric constraints are violated, a lower, suboptimal geometric factor can be selected.
6. In harsh road conditions, thermal effects must be evaluated and accounted by properly sizing the bushing body for optimal heat dissipation. This latter aspect goes beyond the purpose of the present research.

Finally, it is worth noting that this theoretical study does not consider factors like wear, aging or noise, vibration and harshness. These aspects depend on specific features and conditions of each application and therefore, are difficult to extrapolate onto a general methodology.

## 4. Conclusions

The present paper discussed and provided guidelines for selecting rubber materials for bushings as support and isolation elements for vehicle suspensions. In a preliminary phase, the role of the in-wheel machine and the suspension damping were analyzed in the frequency domain. Rubber mounts of viscoelastic material were proposed to support the in-wheel motor mass within the vehicle’s unsprung mass. The mount material was experimentally characterized through isostatic and dynamic tests. Therefore, rheologic parameters were fitted from experiments and included in the suspension through a geometric factor. A sensitivity analysis was performed to understand the role of bushing sizing in the dynamic behavior of the suspension. It was found that road holding is a convex function that can be optimized by selecting specific and similar geometric factors for the bushings that support the in-wheel motor. This optimal selection of the bushing geometric factors led to mount solutions that comply with mechanical and thermal constraints of the design.

Further developments of this work will be aimed at understanding the use of active or semi-active devices to improve vehicle dynamics in vehicles with increased unsprung masses.

## Figures and Tables

**Table 1:** Quarter-vehicle parameters.

Description	Symbol	Value	Unit
Tire stiffness	$k_{1}$	230	kN/m
Wheel mass	$m_{1}$	25	kg
Suspension arm mass	$m_{3}$	11.35	kg
Suspension spring stiffness	$k_{4}$	18	kN/m
Chassis mass	$m_{4}$	187.5	kg

**Table 2:** Elaphe^TM^ in-wheel motor parameters [26].

Model	Mass [kg]	Peak Torque [Nm]	Max. Speed [rpm]	Peak Power [kW]	Continuous Power [kW]
S400	17.6	400	1565	40	23
M700	23	700	1500	75	50
L1500	34.8	1500	1480	110	77
M1100	40	1100	1160	90	70

**Table 3:** Maxwell–Wiechert model parameters for the tested rubber material.

Temperature	$E_{0}$ [MPa]	$E_{1}$ [MPa]	$C_{1}$ [MPa × s]	$E_{1}/C_{1}$ [rad/s]	$e_{rms}$ [MPa]
100 °C	5.9814	0.5261	0.0045	117.86	0.046

## Data Availability

The authors declare to honor the Principles of Transparency and Best Practice in Scholarly Publishing about Data.

## Abbreviations

The following abbreviations are used in this manuscript:

EV	Electric Vehicle
RMS	Root Mean Square
